# From black box to toolbox: Outlining device functionality, engagement activities, and the pervasive information architecture of mHealth interventions

**DOI:** 10.1016/j.invent.2015.01.002

**Published:** 2015-03-01

**Authors:** Brian G. Danaher, Håvar Brendryen, John R. Seeley, Milagra S. Tyler, Tim Woolley

**Affiliations:** a Oregon Research Institute, Eugene OR, USA; b Norwegian Centre for Addiction Research, University of Oslo, Oslo, Norway; c IEQ Technology, Springfield OR, USA

**Keywords:** mHealth, eHealth, toolbox, blackbox, Internet Interventions, Pervasive Information Architecture

## Abstract

mHealth interventions that deliver content via mobile phones represent a burgeoning area of health behavior change. The current paper examines two themes that can inform the underlying design of mHealth interventions: (1) mobile device functionality, which represents the technological toolbox available to intervention developers; and (2) the *pervasive* information architecture of mHealth interventions, which determines how intervention content can be delivered concurrently using mobile phones, personal computers, and other devices. We posit that developers of mHealth interventions will be better able to achieve the promise of this burgeoning arena by leveraging the toolbox and functionality of mobile devices in order to engage participants and encourage meaningful behavior change within the context of a carefully designed pervasive information architecture.

## 1. Background & aims

eHealth interventions have been shown to be effective in encouraging a broad range of health behavior change (e.g., Myung, McDonnell, Kazinets, Seo, & Moskowitz, 2009; Wantland, Portillo, Holzemer, Slaughter, & McGhee, 2004) including, for example, interventions for smoking cessation (Civljak, Stead, Hartmann-Boyce, Sheikh, & Car, 2013; Graham et al., 2011; Strecher, 2007), curbing alcohol consumption (Riper et al., 2011), and managing depression (Griffiths, Farrer, & Christensen, 2010; Spek et al., 2007; Titov, 2011). The promise of eHealth interventions is not limited to Internet interventions delivered on personal computers because it also applies to mHealth interventions delivered on mobile devices (Whittaker et al., 2009). Little is known, however, about, what distinguishes effective from less effective interventions (Webb, Joseph, Yardley, & Michie, 2010).

The burgeoning field of eHealth interventions has focused more on outcomes than on underlying factors and mechanisms – a Black Box approach (Brendryen, Kraft, & Schaalma, 2010; Strecher, 2008). Researchers have proposed several possible remedies to shed more light into the Black Box, including the use of more detailed, standardized reporting of behavior change strategies (Abraham & Michie, 2008), providing comprehensive reporting of the complete intervention rationale along with a description of specific techniques (Bartholomew, Parcel, Kok, Gottlieb, & Fernandez, 2011; Brendryen, Johansen, Nesvag, Kok, & Duckert, 2013; Schaalma & Kok, 2009), and testing new theories of health behavior change (Riley et al., 2011).

There is a growing evidence of the efficacy of mHealth programs to encourage a wide variety of behavior changes, but considerably less research to help inform the intervention designer in choosing the technological tool(s) and devices that will engage participants and help them achieve their desired therapeutic outcomes. It is premature to try to synthesize findings regarding the optimal designs and benefits of mHealth interventions because the field is so new, and the interventions are being used to address so many diverse behaviors/disorders over diverse populations. Instead, in this paper we hope to inform mHealth intervention development by shedding light into the mHealth Black Box by outlining: (1) mobile device functionality -- the technological toolbox available to intervention developers; and (2) the *pervasive* information architecture of mHealth interventions – the way that an integrated intervention can be delivered concurrently using mobile phones, personal computers, and other devices.

## 2. Defining the domain

mHealth interventions include health behavior change interventions that are ostensibly delivered using “…computer devices that are intended to be always on and carried on the person throughout the day” (Riley et al., 2011). mHealth interventions are intended to “…travel through time and space with the participant [whereas] the traditional desktop access method implies [that] participants are tethered to a particular device and are therefore more sedentary (p. 314)” (Turner-McGrievy & Tate, 2014). We also agree with the distinction recommended by Riley et al. (2011) to exclude iPads and other tablets from primary consideration in this paper because they are not typically carried by person throughout the day. Finally, our paper was informed by our adaptation of Ritterband and Thorndike’s (2006) distinction between internet interventions and patient information websites in order to distinguish mHealth interventions from myriad mHealth programs: mHealth interventions are “typically behaviorally or cognitive-behaviorally-based treatments that have been operationalized and transformed for delivery via” *mobile devices*. We also exclude using mobile devices for ecologically momentary assessments except when they are used to inform behavioral interventions.

mHealth interventions have emerged in large part in response to the nearly ubiquitous use of mobile phones: more than 90% of Americans are mobile phone users (Fox & Raine, 2014), with few differences in their gender and race/ethnicity (Lenhart, Purcell, Smith, & Zickuhr, 2010). Trend data indicate that by 2018 almost all Americans will be using smartphones (Smith, 2013). Worldwide use is also very large and rapidly growing, with 2014 estimates of 4.55 billion mobile phone users and 1.75 billion smartphone users (eMarketer, 2014).

mHealth interventions can leverage the fact that mobile phone users typically carry their phones with them throughout the day and even keep them nearby when asleep, making it possible to deliver helpful behavior change content to – and even have interactions with – individuals as they go about their normal everyday lives (Heron & Smyth, 2010; Lenhart, 2010; Patel, Kientz, Hayes, Bhat, & Abowd, 2006). mHealth intervention components can be proactive in that they *reach out* to users to deliver content, prompt interchange, and can deliver persuasive content that encourages behavior change (Fogg, 2007). Heron and Smyth (2010) have described these as ecologically momentary interventions that occur at specifically identified moments in everyday life providing real-time support in the real world.

mHealth interventions can be designed to provide *just in time* support and guidance when most needed (Beale, 2009; USDHHS, 2014; Wikipedia, 2014e). There are at least two ways that mHealth interventions are *just-in-time*. First, intervention content can change based on data obtained during the course of the intervention, as in delivery of text messages that are relevant to a participant’s recent success/problems in managing eating (Intille, Kukla, Farzanfar, & Bakr, 2003) or quitting tobacco (Riley et al., 2011; Ybarra, Holtrop, Bagci Bosi, & Emri, 2012). A technological elaboration of this point can be found in the just-in-time-interventions described by Kumar and his colleagues (Kumar, 2012; Sarker et al., 2014) in which wearable wireless sensors can inform intervention content to enhance successful behavior change (e.g., quitting smoking). The second just-in-time aspect of mHealth interventions involves their immediate accessibility. Because mobile phones are literally within reach they can act as an “as-needed” and available resource, as when coping with a difficult smoking urge the participant could immediately review – and obtain benefit from – helpful content on the smartphone, which might include a personal list of reasons to quit (Ybarra et al., 2012) and/or a relaxation audio (Whittaker et al., 2008).

### 2.1 Taxonomy for defining smartphones

In contrast to current smartphones, early mobile phones did not have a touchscreen, a QWERTY keypad, or the benefits of an advanced operating system. These phones have been described variously as mobile phones having *standard features, feature phones*, and/or *basic phones* (iHeed Institute, 2011), *conventional* (The Nielsen Company, 2013; Wikipedia, 2014c) or *common* (WHO, 2011). These older mobile phones have even been referred to as “dumb phones” to clearly distinguish them from the current generation of smartphones (Wikipedia, 2014c). However, while the label “smartphone” is driven by marketing considerations, an important caution needs to be acknowledged because the “smartness” of today’s phones inevitably will appear much less “smart” when they are compared to the next generation mobile devices. Since smartphones offer so much more functionality than merely making phone calls, the label “mobile device” better captures the breadth of their toolset and the fact that people are able to use them as “converged devices that combine mobility, connectivity, and programmability” (Yuan, 2005).

### 2.2 Taxonomy for defining mHealth

The World Health Organization describes mHealth as a component of the broader category of eHealth (WHO, 2011). A casual Google search using the term will quickly reveal that the mHealth label has been applied to a very considerable breadth of programs and initiatives, including using mobile computing and communications technologies to facilitate care of medical patients (Kotz, 2011) and the use of mobile phones within developing countries to support health workers, collect public health data, and enable health information messaging and helpline services (iHeed Institute, 2011). A taxonomy for mHealth is still emerging and some have questioned whether it will endure as a separate domain because it conceptually and empirically overlaps so considerably with telemedicine, telehealth, and eHealth (Bashshur, Shannon, Krupinski, & Grigsby, 2011). In this paper we have appended the term “intervention” to the mHealth label (yielding “mHealth intervention”) to highlight those programs that are of particular interest to researchers and program implementers of Internet Interventions.

## 3. mHealth toolbox

The specific features available in mHealth interventions depend on the operating system of the mobile device and type of app (if any) being used. Two *first generation* mHealth interventions – text messaging and Interactive Voice Response (IVR) calls – are able to use the basic functionality found on early mobile phones. The toolbox of possible mHealth intervention features available when designed for smartphone users still includes texting and IVR but it is vastly more varied and powerful. For example, smartphone apps can access the Internet, which enables them to access Web-based content, use GPS to track location and provide trip guidance using online maps, and play audio and/or video content. And, rather than a loosely connected collection of tools that reach out to participants, smartphone apps can be designed to offer participants a cohesive multifaceted program to use.

### 3.1 Short Message Service (SMS) text messaging

**Figure 1:**
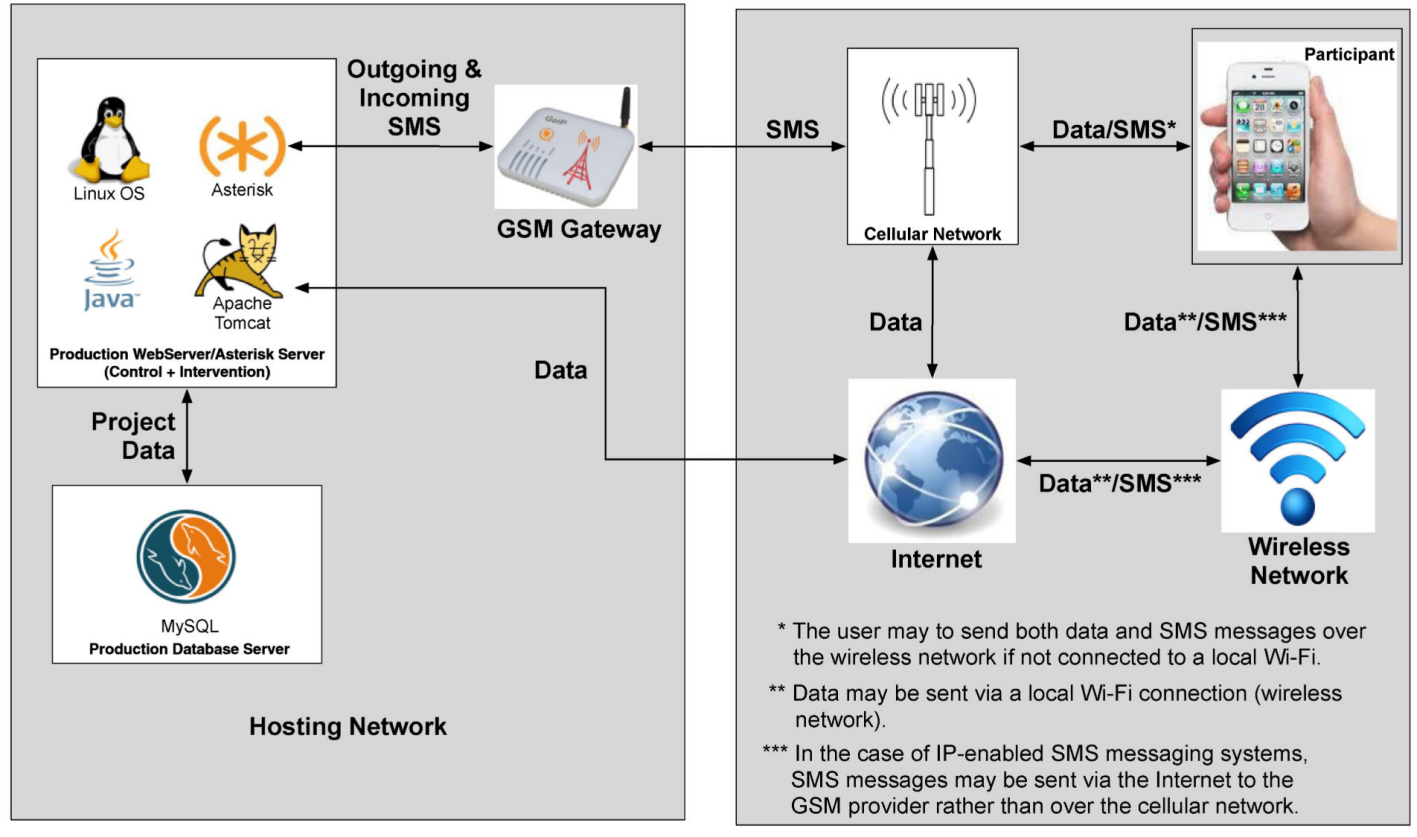
SMS text message infrastructure

SMS messaging involves the delivery of brief text messages that are shared between/among mobile phones. Text messaging can reach all mobile phones irrespective of service provider (Aguilera & Munoz, 2011) and is the most common non-voice use of mobile phones (Smith, 2011), with more than 153.3 billion text messages sent each month in the U.S. (CTIA, 2014). Exchanging text messages can incur charges from the user’s cellular plan, although this cost varies by plan, and unlimited texting is becoming a more commonplace bundled option. It is possible to avoid per-message surcharges altogether by using the device’s proprietary text message functionality available when sender and receiver(s) all use the same mobile device brand (e.g., the iPhone’s iMessage capability). A schematic depiction of the underlying technology required for mHealth interventions to deliver text messages is shown in Figure 1.

Text messages use very brief messages (limited to 160 characters) displayed as an unthreaded top-down manner that are grouped according to the sender phone number. By default, mobile phones typically notify users about the arrival of text messages with an audible alert, a message displayed in the forefront of the screen, and possibly a numeric badge on the text messages app onscreen icon. As a result, text messages can *push* program content to participants in a way that can be relatively difficult to ignore. mHealth programs can be programmed to use text messages as the vehicle for sending users small chunks of program content as well as brief reminders that have a prompting effect (Fry & Neff, 2009). Pre-programmed messages can be scheduled to be delivered at predefined times during the day and in different amounts (numbers of messages) over the course of an intervention. The process can be unidirectional (message arrive with program content) as well as bi-directional (text messages ask questions of users whose simple text message replies can be used by the program to tailor the delivery of program content). There is a growing research track record showing beneficial effects from using automated text messaging for health behavior interventions (Fjeldsoe, Marshall, & Miller, 2009; Fjeldsoe, Miller, & Marshall, 2010; Patrick, Griswold, Raab, & Intille, 2008). Meta-analyses (Fjeldsoe et al., 2009; Fjeldsoe et al., 2010; Krishna, Boren, & Balas, 2009) have documented text message interventions that have been used for diabetes self-management (Franklin, Waller, Pagliari, & Greene, 2006; Yoon & Kim, 2008), eating disorders (Robinson et al., 2006; Shapiro et al., 2010), physical activity (Prestwich, Perugini, & Hurling, 2009), and tobacco cessation (Brendryen, Drozd, & Kraft, 2008; Brendryen & Kraft, 2008; Free et al., 2009; Rodgers et al., 2005).

The therapeutic benefits associated with text messages will likely vary based upon their content and tone, their bi-directional sharing of content, and the schedule and density of their delivery. For example, delivering too many messages may well be intrusive, annoying, and thus unhelpful. But length limitations can cause text messages to read like fortune cookie messages that have extraordinarily limited opportunity for empathy, nuance, and engagement. All of these features represent important areas for research inquiry.

### 3.2 IVR automated calls

IVR programs delivered on mobile phones essentially involve sending a recorded phone message in an automated calls. Because of this simplicity, these IVR programs function quite well on standard feature mobile phones, and, of course, also on land-line phones. Similar to text messaging, calls programmed for delivery by the IVR system arrive with an audible alert (an incoming phone call ringtone). Users are then able to listen to program content (unidirectional audio content delivery). Some IVR systems enable users to reply to questions presented in a call they have received (bi-directional content). Many mobile phone users may find it difficult to use their phone’s keypad for data entry while they remain engaged in the phone call. More sophisticated IVR systems are able to interpret simple verbal responses thereby avoiding this usability barrier.

Just as with text messages, IVR calls proactively *push* content to participants. Many health behavior change interventions and medical support programs have successfully used IVR systems (Abu-Hasaballah, James, & Aseltine, 2007; Bartholomew et al., 2011), including programs for physical activity (Pinto et al., 2002), healthy eating (Delichatsios et al., 2001; Estabrooks et al., 2009; Estabrooks & Smith-Ray, 2008; Piette, 2000, 2002), medication refills (Reidel, Tamblyn, Patel, & Huang, 2008), caregiver support (Mahoney, Tarlow, & Jones, 2003), depressive symptoms (Osgood-Hynes et al., 1998), delivery of ambulatory care (Oake, Jennings, van Walraven, & Forster, 2009), diabetes self-management (Piette, 2000), and smoking cessation (Ramelson, Friedman, & Ockene, 1999; Reid, Pipe, Quinlan, & Oda, 2007). When IVR calls are programmed for receipt on a mobile phone they become part of the possible toolset for mHealth interventions (Brendryen et al., 2008; Brendryen & Kraft, 2008).

### 3.3 Smartphone apps

While there is increasing discussion regarding the taxonomy of the term *apps* vs. *applications* (Lewis, Boissaud-Cooke, Aungst, & Eysenbach, 2014) and apps classification schemes (Wang et al., 2014), we consider three general types of smartphone applications: native (operating-system-based) apps, Web apps, and hybrid apps that combine features found in both native and Web apps (Nielsen Norman Group, 2014).

*Native apps* use the sophisticated features and functionality made available through the mobile phone’s operating system (e.g., iOS for iPhones as well as Android). For example, they can use GPS-derived location, the system calendar, system alarms, and other notifications. Some native apps can function effectively without persistent or live Internet access. Because native apps use data available through the mobile phone’s operating system, they generally must adhere to various design and review requirements of the company overseeing the operating system, (Apple, 2013; Google, 2014), and be downloaded via app stores hosted by the smartphone’s manufacturer. One exception to this rule involves “enterprise deployment” which permits the provisioning of *in-house* apps by large organizations directly to end-users while bypassing the app store altogether (Apple, 2014). For example, enterprise deployment can be used to deliver custom apps by corporations to their employees, health care organizations to their patients, and even research organizations to distribute mHealth interventions to a designated audience of users according to custom rules (e.g., treatment allocation, research assignment), something not as easily accomplished using app stores.

*Mobile browser or Web apps* are essentially websites that are delivered using the smartphone’s browser. The selection of content and the manner in which it is displayed are controlled by the logic contained in a program hosted on a remote server (server-side). As with desktop-based Web interventions, mHealth interventions using mobile Web apps are able to incorporate sophisticated levels of interactivity, tailoring, and engagement tracking. Compared to a native app, mobile browser apps don’t require review and distribution by a smartphone manufacturer. Because they do not require different programming in order to fit the functionality of different operating systems, Web apps can be easier and less costly to develop than native apps when mHealth interventions are to be implemented on multiple smartphone devices. Because their content and program rules are controlled by a remote server, mobile browser apps require persistent access to the Internet. Moreover, mobile browser apps tend to be somewhat slower and less responsive than native apps.

*Hybrid apps* incorporate features and functionality found in native apps with the versatility and efficiency associated with using mobile browser apps. They are able to display program content using a browser that is embedded within the native app itself rather than simply using the smartphone’s browser. As a result, hybrid apps can offer a more tightly integrated environment (envelope) than mobile browser apps. They can provide multiple tools some of which draw upon the *intelligence* and data that are only available from other native (built-in) smartphone features.

The choice about what type of app is best for mHealth interventions depends upon analysis of available programming/development resources, the need to access data from the native operating system, the importance of delivering a very tightly integrated intervention, and ease of distribution. Interventions that use websites – whether delivered within Web apps or hybrid apps – tend to be reactive; they wait for users to visit (Brendryen et al., 2010; Heron & Smyth, 2010; Riley et al., 2011). This passivity can be balanced with other mHealth intervention components (e.g., text messages) that *push* program content to participants in salient ways that the user is more likely to notice and use.

### 3.4 Email

Email fits within the broader category of mobile phone text notification tools (Mohr, Schueller, Montague, Burns, & Rashidi, 2014). Similar to text messaging and IVR calls, email proactively pushes content to mHealth intervention participants. By default, the arrival of email is far less salient because it may not have an audible or visible signal. In these circumstances, email may require participants to seek it out at their own initiative. It is possible to increase salience by asking program participants to change their default settings in order to assign audible sounds or visible alerts that *announce* its arrival. Automated email has been found to be a helpful feature that is frequently included in eHealth interventions to increase participant engagement and program efficacy across a wide variety of problems (e.g., Civljak et al., 2013).

### 3.5 Program content display using onscreen text

mHealth smartphone apps display some portion of their content as paragraphs of onscreen text. Browser-based mHealth interventions can be designed to automatically rearrange and reduce the size of program text for different screen sizes (personal computer, mobile phone, large-scale mobile phone or phablet, and tablet) and display orientation (portrait vs. landscape). These responsive (Wikipedia, 2014g) or adaptive (Wikipedia, 2014a) scripting approaches enable Web applications to automatically identify the user’s device or the attributes of the user’s device (e.g., browser viewport size and screen resolution). This information is then used to tailor the types of content displayed as well as the manner in which it is displayed – thereby resulting in a distinct user interface for each device (Nielsen & Budiu, 2013). Although conceptually attractive, this *one-size-fits-all* browser-based approach can be quite complicated to program, and many times underlying program logic needs to be redesigned and not just the display of content. The delivery and display of mHealth intervention content will inevitably require the designer to create rules to define which content to display as well as how it should be formatted.

### 3.6 Text notifications

**Figure 2:**
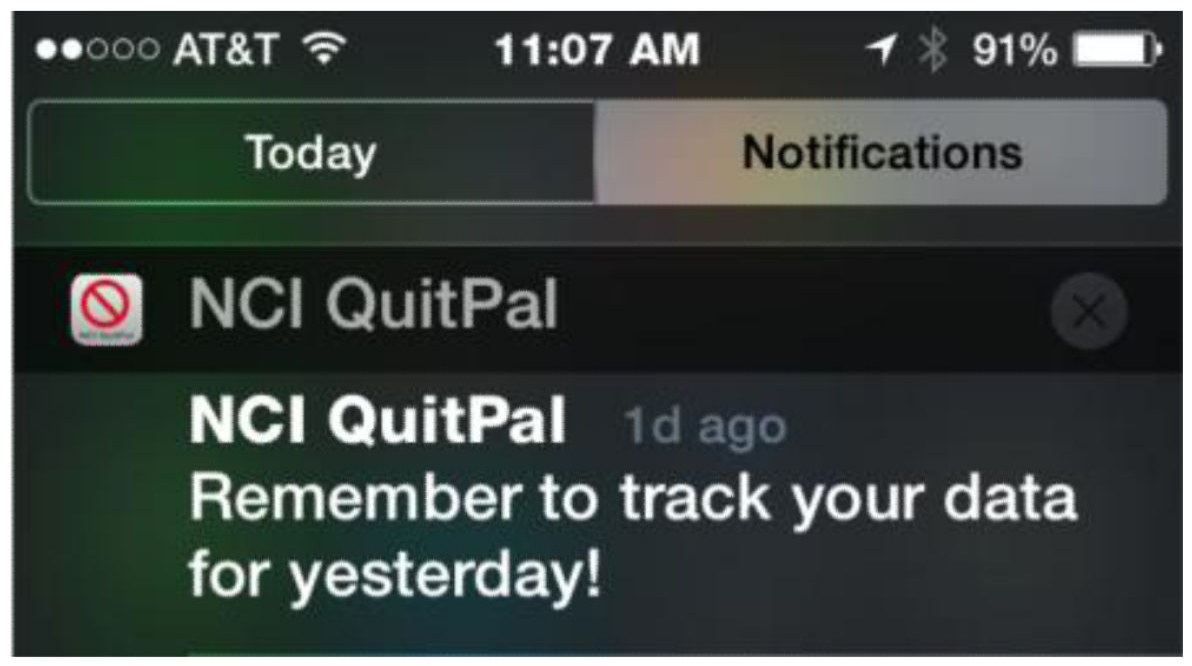
Example of text notification feature of the National Cancer Institute’s *QuitPal* iPhone-based smoking cessation intervention app (NCI, 2014)

mHealth apps can also proactively push intervention content to participants by displaying salient text notifications with alerts, mHealth intervention apps can use a broader set of tools to notify participants in various ways. For example, they can display text onscreen which occurs with a corresponding audible alert sound (see Figure 2). Rather than being constrained to unthreaded top-down “conversation” grouped within the sender’s phone number, custom text notifications can be displayed and catalogued in ways that are easier for the user to find and review – thus potentially increasing their impact. Moreover, when tapped, custom text notifications can enable users to access specific content available within a native app – a degree of integration that cannot be accomplished using text messages.

### 3.7 Audio and video notifications

Smartphone-based mHealth interventions can be designed to include audio content from “simulated places and people” (Mohr, Burns, Schueller, Clarke, & Klinkman, 2013), avatars or even mobile health counseling agents (Bickmore, Mauer, & Brown, 2009). Asking participants to have human-like audio interchanges with their mobile phones seems increasingly acceptable -- and possibly feasible – based upon the rapid widespread use of intelligent personal assistants (conversational agents) available on current smartphones including *Siri* (iOS) (Wikipedia, 2014h), *Cortana* (Microsoft) (Warren, 2014), *Google Now* (on multiple smartphone OS) (Wikipedia, 2014d), as well as assistants from software companies (e.g., *Nina* from Nuance (Nuance, 2014)). Users are increasingly using voice commands to interact with features of their smartphones using these digital personal assistants. And, in some cases, to being guided by verbal and/or text advice from their smartphone’s digital assistant.

Similarly, mHealth intervention apps can deliver video content to participants. Examples include the use of video testimonials (Whittaker et al., 2008; Whittaker et al., 2012), and using animated presentations.

### 3.8 Recording pictures, audio, and video

**Figure 3:**
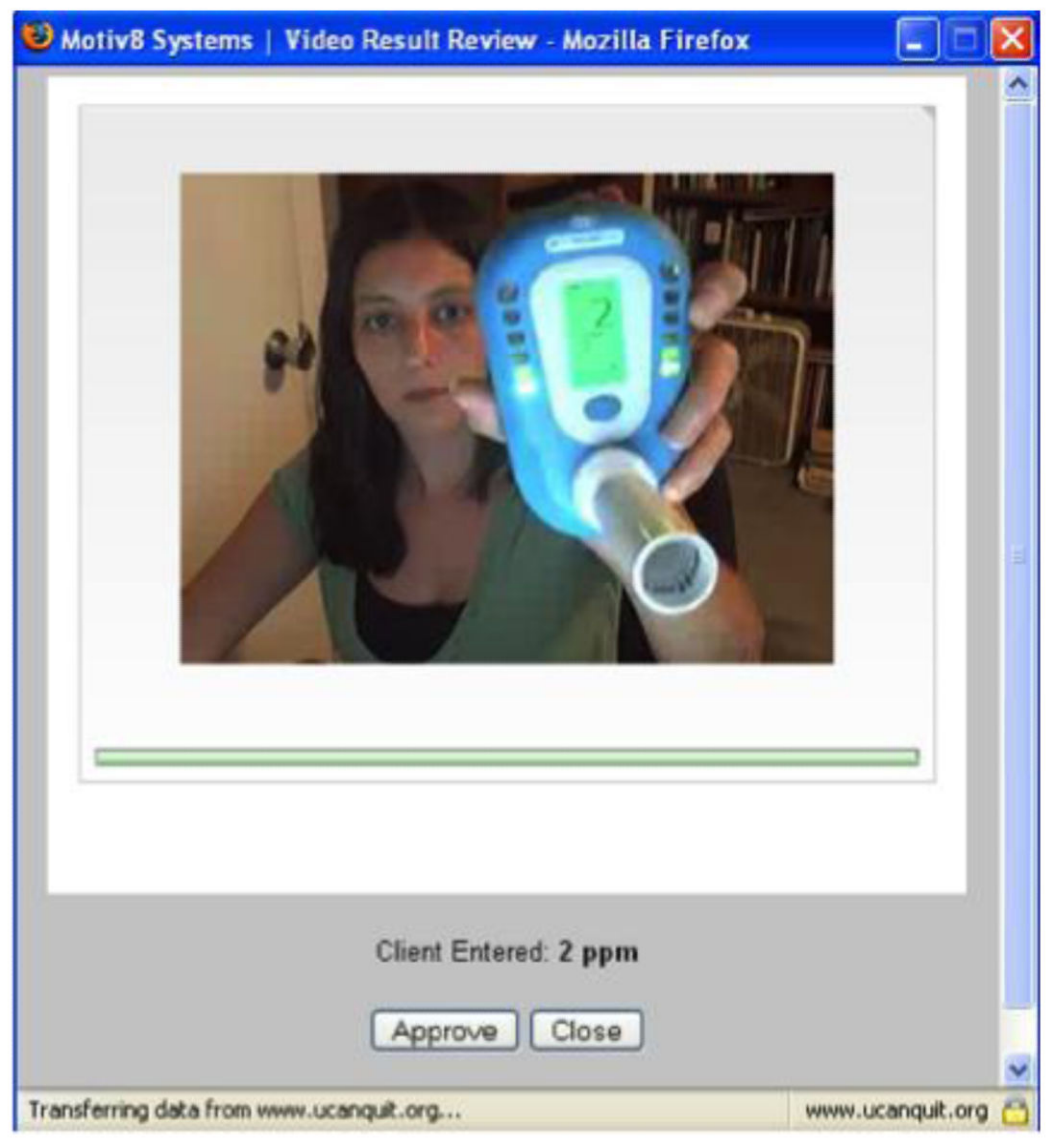
Example of how video of a Carbon Monoxide meter can be used to confirm self-reported smoking abstinence (used with permission from R. Dallery, 2014).

Designers of mHealth interventions can draw upon the smartphone’s built-in tools to provide key data. For example, participants in a “photovoice” intervention (PhotoVoice, 2014; Wikipedia, 2014f) used their smartphone camera to accomplish an assigned task of taking pictures of surroundings and experiences that reflected their weight-related concerns (Woolford et al., 2012). One mHealth intervention measured participant meal portion sizes using images captured by participant smartphone camera (Six et al., 2010). Other studies have asked participants in an Internet cessation study to use their computer’s video capability to show a reading from a Carbon Monoxide meter as a way to validate their self-report – an approach that could be easily adapted for a smartphone intervention (Dallery, Glenn, & Raiff, 2007; Dallery, Raiff, & Grabinski, 2013) (See Figure 3). Similarly, the smartphone’s microphone can be used to record conversations with coaches/counselors for subsequent review (Luxton, McCann, Bush, Mishkind, & Reger, 2011). Finally, the tempo of smartphone-delivered music has been tailored to encourage participants to increase their physical activity (Liu et al., 2008).

### 3.9 Sensor functionality

The use of sensors in mHealth interventions is in its infancy. There are a growing number of published descriptions and initial evaluations of innovative sensor-enhanced interventions already appearing (e.g., Clifton, Clifton, Pimentel, Watkinson, & Tarassenko, 2013; Cowie, Chronaki, & Vardas, 2013; Kumar et al., 2013; Moore et al., 2007; Scully et al., 2012; Warmerdam et al., 2012) – although a number of the more sophisticated current generation sensors tend to be bulky contraptions attached to the user’s body rather than built into a smartphone. Nonetheless, sensors offer the promise of being able to provide the unobtrusive capture of personally relevant information that may be able to detect when a particular intervention strategy might be most helpful (Mohr et al., 2013). In the U. S., Kumar and his colleagues (Kumar, 2012; Sarker et al., 2014) and his NIH-funded center (MD2K, 2014) are in the early phases of developing just-in-time-interventions that use behavioral and situational data derived from wearable wireless sensors to inform intervention content as a way to significantly enhance behavior change (e.g., quitting smoking). A parallel initiative involving European researchers (Guiry, van de Ven, Nelson, Warmerdam, & Riper, 2014; Warmerdam et al., 2012) has examined the use of attached sensors within the context of an eHealth depression intervention that also included a smartphone adjunct (ict4depression, 2014).

Mobile phone sensors can capture data using devices external to the mobile phone such as a blood pressure monitor, wearable devices like smartwatches and wristbands, as well as Internet-based data available to mHealth participants like relevant weather and traffic. mHealth interventions could consider using data from sensors embedded in the mobile phone (e.g., data on the calendar and time of day from the calendar, location/proximity from GPS, movement via the accelerometer, step counter, sound from the microphone (Kumar, 2014; Mohr et al., 2014). For example, a recent mHealth intervention (Addiction-Comprehensive Health Enhancement Support System or A-CHESS) that used the smartphone GPS system to detect when a participant was within a certain distance of a high-risk location (e.g., a bar visited in the past) in order to send a text message alert (Gustafson et al., 2014). Other examples using GPS to assist alcohol treatment also have emerged (Dulin, Gonzalez, & Campbell, 2014). The distinction regarding the source of sensor data (i.e., data derived from sensors within the mobile phone vs. from sources external to the mobile phone) blurs as device-based sensors feed as well as read from external devices, including cloud-based data sources and wearable devices and possibly even devices placed strategically in our environment (geofencing). Although the accuracy, usability, and battery consumption of current sensors need to be improved, the use of sensors by mobile devices to track myriad user data can be expected to dramatically improve over time – and increase in value to mHealth interventions.

### 3.10 Discussion forums and blogs

mHealth applications sometimes use discussion forums (web blogs or forums) designed to enable program participants to interact with each other, sharing their stories and support (Gustafson et al., 2014). This feature can be particularly helpful if sources of support are difficult to find and if lack of anonymity and privacy are key barriers to otherwise seeking support (as in cases that involve considerable social stigma). As peer-to-peer social media tools like Facebook and Twitter have become ubiquitous, it may be superfluous for mHealth interventions to try to design their own tools that attempt to mimic this functionality. However, there may still be reasons to wrap a discussion forum within the *envelope* of an mHealth app, as when there are extreme concerns about participant privacy and/or when associated with higher risk situations (e.g., suicidal behavior) that might well require the ongoing review of forum activities by a trained moderator. Moreover, it is difficult to carefully measure the extent to which participants use Facebook and Twitter because unobtrusive measures of participant engagement are typically limited to use of features directly provided by the mHealth app.

### 3.11 App-specific design and engagement activities

**Figure 4:**
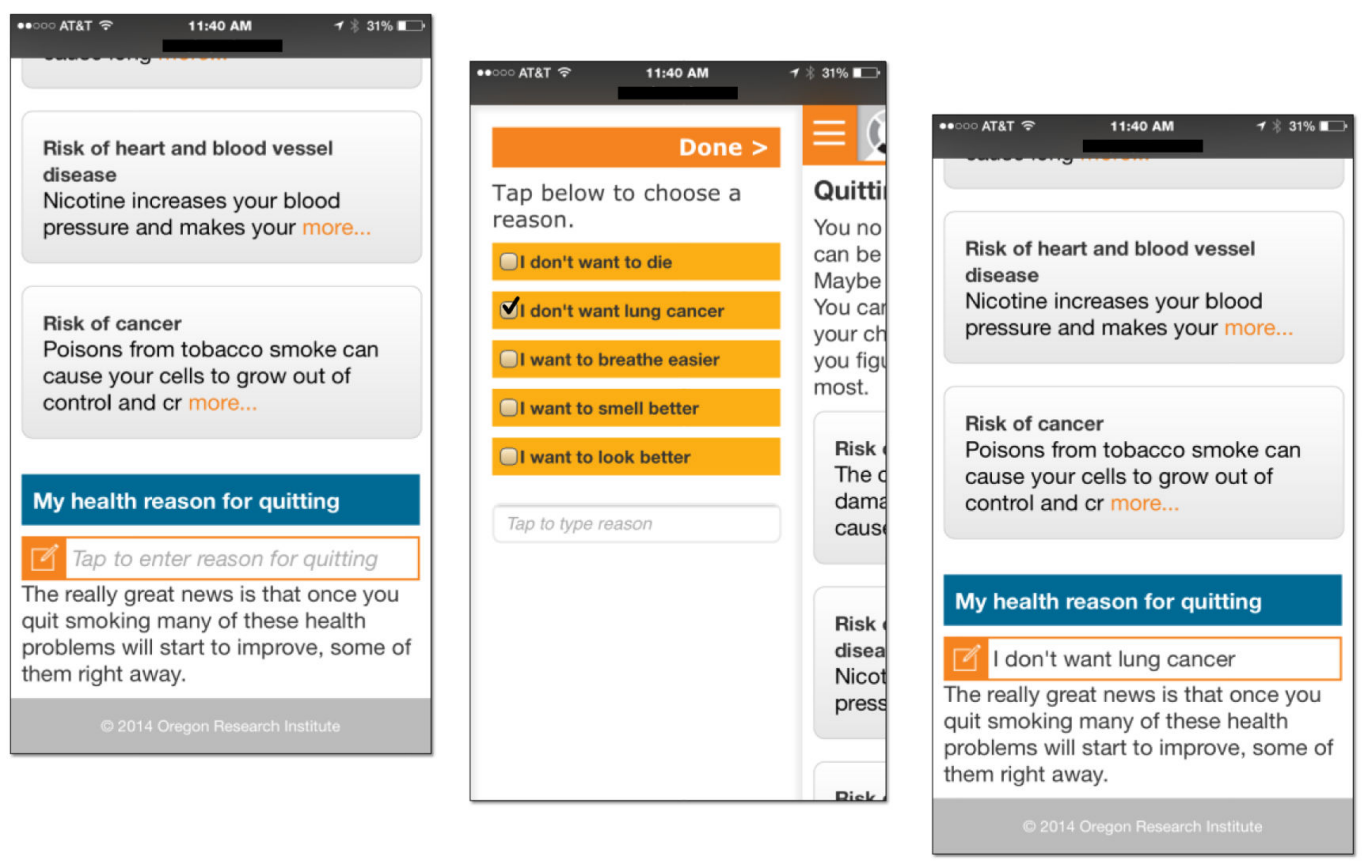
Draft screenshots (My health reasons for quitting) illustrating an interactive Lists activity (see Table 1) excerpted from our mHealth smoking cessation browser app. Tapping field in left screen triggers popup to appear (center screen), which enables user to choose from fixed list or type in text, which then causes popup to close and reveals personally-relevant health reason for quitting (right screen).

The manner in which the mHealth intervention app developer takes advantage of the mobile device’s functionality can encourage participant engagement. At a relatively simple level, this can include following interface guidelines unique to the smartphone so that the user can build on standard appearance and functionality of what is presented on-screen (Apple, 2013; Google, 2014). Examples include ensuring that apps do not include an exit button, that they start back up where the user left off, and they respond to changes in device orientation (landscape or portrait). In addition, mHealth intervention app activities will probably embody variations of the same participant engagement activities used in eHealth interventions designed for personal computer-based interventions – see Table 1 adapted from Danaher et al. (2013). A sample of the Lists activity is illustrated in Figure 4.

Another just-emerging category of activities not included in the table but nonetheless deserving consideration involves serious games that are designed to use smartphone tools to be engaging while encouraging “players” to change their behavior. For example, a commercial Android app named “Dance! Don’t Fall” asks users to wear their smartphone on their lower back to enable the device’s accelerometer to track dance steps designed to prevent falls and promote exercise at home (Kerwin, Nunes, & Silva, 2012; Silva et al., 2013). Another Android game app called “SmartCAT” is a smartphone app for childhood anxiety treatment designed to work with a therapist portal (Pramana, Parmanto, Kendall, & Silk, 2014; University of Pittsburgh, 2013).

## 4. Pervasive information architecture

### 4.1 Information architecture of interventions delivered on personal computers

In our original discussion of information architecture and health behavior change websites (Danaher, McKay, & Seeley, 2005) we focused on the design of individual websites that we assumed would be available on desktop computers. In that report we delineated a number of information architecture designs that differed in the way that users were able to view content:

- a *tunnel design* guides users through a step-by-step process to allow program webpages to be accessed in a particular order to improve the chances of achieving a goal that is measurable and consistent. Upon entering a tunnel the user accepts a lowered degree of autonomy, as if the mHealth program becomes a demanding coach who *pushes* the participant to realize his/her success (Fogg, 2003).
- a *matrix design* enables users to explore available content without any programmatic constraints.
- a *hierarchical design* organizes information in a *top-down* manner so that users can choose to *drill down* in order to access increasingly detailed content.
- a *hybrid design* combines elements of the tunnel, matrix, and hierarchical designs to enable users to access program content according to *workflow rules* (Mohr et al., 2014) that govern, for example, the amount, order (timing), and detail of program content.

### 4.2 Combining mHealth with interventions delivered on personal computers

At a conceptual level, the four information architecture designs could simply be extrapolated to fit mHealth interventions. But the paradigm of an intervention delivered exclusively on a personal computer fails to adequately capture the *push* and *pull* features of mHealth interventions. For example, the emerging mHealth paradigm is shaped by what usability experts have described as mobile phones’ “impoverished user experience” with “…tiny screens, slow connectivity, higher interaction cost (especially when typing, but also due to users’ inability to double-click or hover), and less precision in pointing due to the “fat finger” problem.” (p. 34; Nielsen & Budiu, 2013). Constrained screen size and interactivity point to the need to design mHealth interventions that have less complicated content and require less interaction from users. One way this might be accomplished is to intelligently ration how much and what kind of content such interventions should include. For example, it might be best for them to focus on content that has the greatest potential impact in terms of changing certain behaviors (e.g., motivational messages, behavioral prompting, self-monitoring and charting, and/or other proactive action-focused program content) using the toolset features and functionality described in this report (e.g., text messages, IVR call prompts, notifications).

Secondly, simply porting a personal computer design to the smartphone would potentially ignore the *push* features available when using text messaging, IVR calls, or text notification in mHealth interventions. These features can encourage greater participant use of the intervention, and provide a sense of program vitality and responsiveness.

Third, hybrid eHealth interventions combine mHealth program components with Web-based interventions accessed on a personal computer. In these scenarios, mobile phones could push the delivery of *just-in-time* content designed to promote interaction, increase motivation, challenge dysfunctional beliefs, and provide cues to action (Klasnja & Pratt, 2012; Webb et al., 2010). With its greater screen space and a more usable interface, the personal computer component could provide rich content with enhanced interactivity and multimedia features, charting, long-term access to resource descriptions, etc. For example, a recent mHealth depression intervention was delivered using this type of hybrid approach: “…monthly text messages directed participants to a mobile website. This website (available only to recognized participant mobile phone numbers) provided a summary of the key messages, information on how to get more help, and a downloadable relaxation audio. New videos were posted on the website monthly. Ringtones, wallpaper images, and music downloads were linked to the mobile website” (Whittaker et al., 2012).

Just as with adjunctive eHealth interventions (Danaher & Seeley, 2009), a variation on the hybrid model involves mHealth interventions that are combined with non-technology treatment adjuncts including, for example, face-to-face treatment, telephone-based interventions (Glasgow, 2007; Glasgow, Christiansen, Smith, Stevens, & Toobert, 2009), and pharmacotherapy (Brath et al., 2013; McGillicuddy et al., 2013). Moreover, a therapist portal on a personal computer could be used to increase the integration of an mHealth intervention with face-to-face treatment (e.g., Pramana et al., 2014) possibly by enhancing the supportive accountability associated with therapist/coach contact/feedback (Mohr, Cuijpers, & Lehman, 2011).

### 4.3 Pervasive information architecture of mHealth interventions

**Figure 5:**
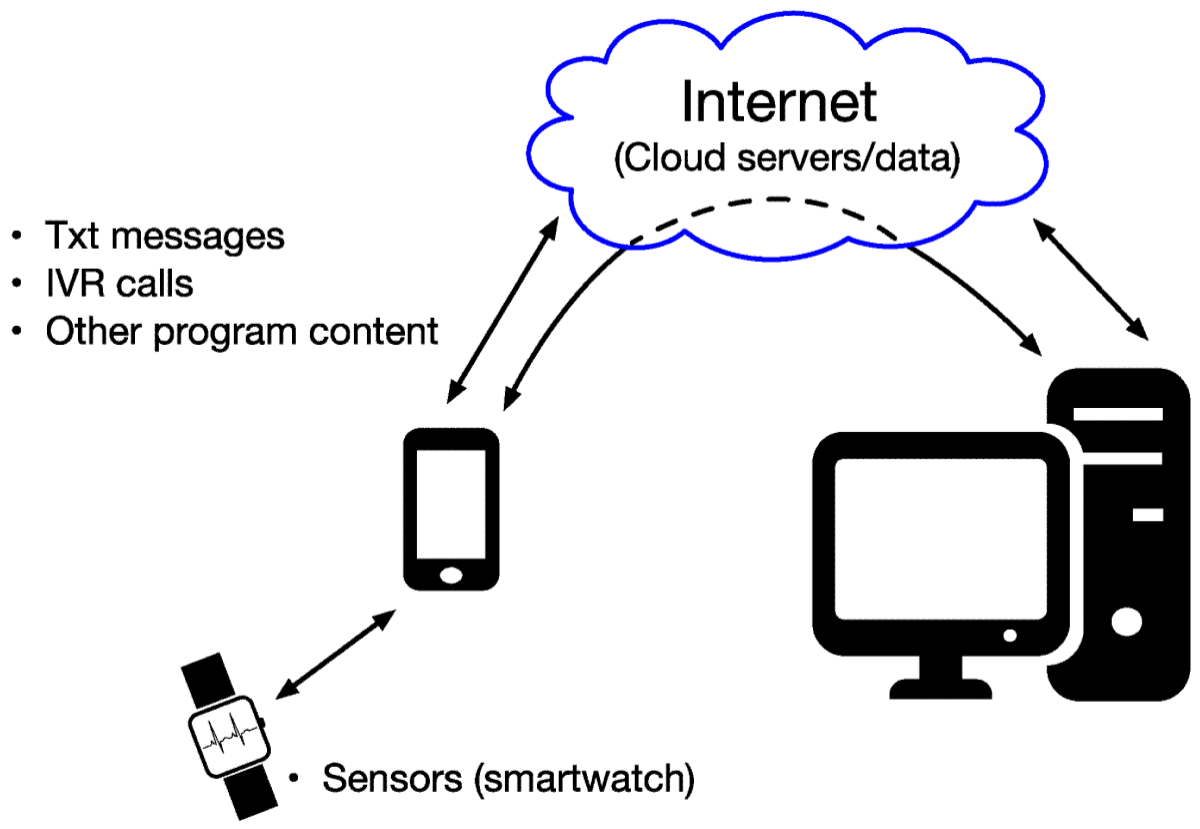
Schematic of data shared across multiple devices in a hybrid eHealth/mHealth intervention that warrants consideration of pervasive information architecture

The emergence of hybrid eHealth interventions delivered on both personal computers and mobile devices requires a more sophisticated view of information architecture – one that coordinates the interplay between different devices. This expanded view ubiquitous computing (“ubicomp”) underscores the need for “pervasive information architecture” that takes into consideration the broader interplay of devices and the way that they share data/information (Greenfield, 2006; Resmini & Rosati, 2011). The simpler information architecture that informs the top-down and left-right flow of websites on personal computers needs to be considered within the added context that designs the interplay of content delivered on other devices (see Figure 5). For example, it is essential that the hybrid design enables the participant to benefit from a single coherent, tightly-integrated experience: “…the lack of coordination between communicating or mutually-supporting channels is bound to affect the whole process. When multiple interactions are designed as unstructured and unrelated, but are in fact perceived as one single experience by the user…structural gaps and behavioral inconsistencies are common and unavoidable, and the sheer cognitive load and awkwardness of switching back and forth between noncommunicating and apparently diverse touch points hampers the final user experience.” (p. 43, Resmini & Rosati, 2011).

To achieve the single user experience, the framing of the messages should be similar and thus familiar across devices. And the data the user provides to the intervention as well as the information provided by the program to the participant must be immediately populated across all related devices. Similarly, although content may be delivered on each device using a distinct schedule or calendar – probably related to the steps involved in making health behavior changes – the user should perceive the program as having a single schedule tailored to fit their needs. Seamlessness requires extremely tight integration. Similarly, Resmini and Rosati (2011) offer five heuristics to inform the design of unified, integrated interventions:

1. Place-making—help users find their way across complementary digital, cross-channel, and even physical environments.
2. Consistency—provide a model that works for the program’s purpose and benefits end-users across different media, channels, and time.
3. Resilience—adapt program content to fit user needs, preferences.
4. Reduction—present program content to users in a way that is simpler and more usable than the underlying complexity of program design.

## 5. Discussion

This report presents a current snapshot of mHealth intervention issues. Some of these issues focused on the technology available in the most current generation of mobile phones that can be brought to bear on mHealth interventions. Also reviewed was how device functionality maps onto the essential strategies that can help encourage behavior change: the push/pull impact, the use of behavioral prompts via email, IVR, and notifications (text, pictures, audio/video), the possible integrating of data available from internal and external sensors and from within the native operating system data, etc. Next we examined the increasingly important contributions of the field of pervasive information architecture that informs the way to take best advantage of the fact that people tend to have – and selectively use – multiple technology tools.

The use of technology to encourage behavior change using mHealth interventions is in its infancy, and it is ripe for both creativity and empirical examination. For example:

- How practical (conceptually, behaviorally, financially) is it to develop, deliver, and maintain intervention content in one medium/channel versus others?
- Under what circumstance might an intervention use only a single channel (e.g., a mobile device) to encourage health behavior change?
- What barriers to efficacy might point to the need to deliver intervention content on a personal computer versus a mobile device? How would a staged-model apply to choosing one approach first followed by the second approach if unsuccessful?
- Is it important to assess the extent to which individuals assigned to an mHealth intervention actually use a mobile device to access that content? (See Turner-McGrievy and Tate (2014) and The Nielsen Company (2013)).
- Should participants be prevented (or actively discouraged) from using mHealth content on their personal computers?
- To what extent are tablets used like personal computers or mobile devices? Their screen size is larger than smartphones yet their interactivity still similarly constrained. Moreover, tablets tend not to be used in a portable manner during everyday routines.
- Is there a benefit to scheduling the delivery of a series of brief, interrelated text messages – e.g., text message adaptations of sequential Burma-Shave messaging (Wikipedia, 2014b) – rather than as independent chunks of content?
- In an effort to be sensitive to respondent burden, should longer and more complex assessments associated with research trials be available only via personal computers rather than on mobile devices? Because of their brevity, could screening assessments be on both?
- Given that the boundaries between/among devices are becoming more permeable (e.g., Apple’s Yosemite and iOS 8 operating systems now deliver text messages and phone calls to personal computers as well as iPhones), does this foreshadow broader platform reach for text messaging and other notifications in designing hybrid eHealth interventions?
- Are there certain mHealth tools (e.g., video, digital assistants, tailored text messages, etc.) that are particularly helpful in terms of strengthening the working/therapeutic alliance with the intervention, which results in enhanced engagement and improved outcomes?
- When should interventions be designed/delivered only on smartphones versus delivered with other devices (e.g., personal computers) using a hybrid model? How does therapist/coach contact enhance participant engagement in mHealth interventions and affect outcome?
- How can data and results from mHealth interventions populate electronic medical records (EMRs)?

Clearly, additional research is required to determine the proper roles for mHealth and personal-computer-based intervention components. Because the trend towards pervasive smartphone usage seems inexorable and the attraction to use smartphones and other mobile devices for behavior change interventions is growing in parallel, intervention designers will need to take care to avoid simply porting over intervention designs intended for personal computer users while using multiple channels and devices. By leveraging the ever-increasing toolbox functionality of mobile devices and addressing the context of pervasive information architecture, we believe that mHealth intervention developers will be better able to achieve the promise of this burgeoning arena by engaging participants and encouraging meaningful behavior change.

## Figures and Tables

**Table 1:** Engagement activities for consideration in mHealth intervention apps

Activity	Function	Examples
Lists	Add personal content using lists or typing in own content	To list pleasant activities, supporters, reasons for wanting to change, high- tension situations, warning signs
Expand-collapse content	Explore additional detail on topics of interest.	To explore FAQs, Myths & Facts, etc.
Wizards tool	Multi-step interaction that builds towards a strategy	To encourage goal selection or identify lessons learned (e.g., a lapse/relapse in order to avoid slips in the future)
Practice change activities	*Homework* tasks to be accomplished in normal routine	To track use of relaxation methods to manage stress, or to anticipate and savor activities
Behavior tracking	Capture and display participant data over time designed to encourage self-monitoring, show patterns and progress	To track and chart smoking status, mood ratings, pleasant activities
Videos	To provide content and encourage use of recommended strategies	To deliver content from program host, testimonials from others describing experiences, ways to overcome barriers, revise strategies, plan for the future
Animated tutorials	Explain underlying models for change	Use animation to show downward spirals for mood and urges as well as how they can be interrupted at critical choice points

## Highlights

- mHealth interventions, a subset of the broader realm of eHealth interventions, are growing rapidly in number and are being used as stand-alone tools for behavior change as well as adjuncts to other interventions.
- mHealth interventions can leverage smartphone (mobile device) functions to encourage behavior change.
- The content (including mobile device tools and engagement activities) and information architecture can borrow from more established guidelines for interventions delivered via personal computers. But mHealth intervention developers need to adapt their approaches to “fit” the strengths, constraints, behavioral purposes, and pervasive information architecture of the mHealth paradigm.
